# Autophagy in ischemic stroke: pathophysiology, therapeutics, and challenges ahead

**DOI:** 10.3389/fstro.2026.1769395

**Published:** 2026-07-20

**Authors:** Tshibambe N. Tshimbombu, Arsene Daniel Nyalundja, Gates Mulume Iragi, Josué Aganze Mwambali, Samira Braimah Shardow, Melissa Ewurakua Amoako, Kyle E. Thurmann, Daniel I. Gonzalez, Paige Banyas, Judea Wiggins, Supreet Kaur

**Affiliations:** 1 Department of Neurology, Barrow Neurological Institute at St. Joseph's Hospital and Medical Center, Phoenix, AZ, United States; 2 Center for Tropical Diseases and Global Health, Faculty of Medicine, Université Catholique de Bukavu, Bukavu, Democratic Republic of Congo; 3 Faculty of Medicine, Université Catholique de Bukavu, Bukavu, Democratic Republic of Congo; 4 School of Medicine, University of Ghana, Accra, Ghana; 5 School of Medicine, Creighton University, Phoenix, AZ, United States

**Keywords:** autophagy, ischemia-reperfusion injury, ischemic stroke, neuroprotection, therapeutic targets

## Abstract

Autophagy is a fundamental cellular homeostatic process that exerts a dual, context-dependent influence on the pathophysiology of ischemic stroke. Functioning as both a neuroprotective survival mechanism and a neurotoxic pathway, autophagy presents a complex therapeutic challenge as well as a potential target for molecular intervention. This narrative review synthesizes preclinical and emerging clinical evidence to summarize key mechanisms regulating autophagy in ischemic injury, evaluate therapeutic strategies, and identify promising molecular pathways and druggable targets for translational development. In the early ischemic phase, moderate autophagic activation facilitates neuronal survival by clearing damaged mitochondria and protein aggregates, thereby reducing oxidative stress and modulating neuroinflammation. This protective response is primarily mediated by regulators such as Beclin-1, the conversion of LC3-I to LC3-II, and the energy-sensing AMP-activated protein kinase pathway. Conversely, sustained or excessive autophagy, particularly during late-stage reperfusion, exacerbates neuronal injury through impaired lysosomal fusion, autophagosome accumulation, and the triggering of autophagic cell death and ferroptosis. Preclinical evidence highlights a critical Goldilocks zone of activation, suggesting that therapeutic success hinges on maintaining autophagic flux within narrow physiological limits. Advancing these therapies into clinical practice requires precise spatiotemporal modulation, potentially as an adjunct to mechanical thrombectomy, as well as the development of robust, real-time biomarkers. A comprehensive understanding of the molecular and genetic determinants of autophagy, including sex-specific responses, is essential to bridge the translational gap and establish autophagy as a viable target for precision stroke medicine.

## 1 Introduction

The global disease landscape has been shifting, with noncommunicable diseases emerging as the primary drivers of mortality and morbidity. Among these diseases, stroke remains one of the leading causes of death and long-term disability worldwide, accounting for over 7 million annual fatalities and 160 million disability-adjusted life years (Feigin et al., 2025). Beyond the clinical impact, the economic impact is profound, with estimated global costs exceeding $891 billion annually, a figure projected to nearly double by 2050 (Feigin et al., 2025).

Despite advances in recognition and diagnosis, the efficacy of current clinical interventions remains constrained by narrow therapeutic windows, systemic disparities in care access, and the risk of procedural complications (Chen and Jin, 2023; Chukwudelunzu and Mbonde, 2024; Mo et al., 2020). These limitations necessitate a paradigm shift toward understanding the endogenous molecular mechanisms underlying ischemic injury. Modulating cellular processes such as neuroinflammation, oxidative stress, and programmed neuronal death offers a promising frontier for mitigating cerebral damage and enhancing long-term functional recovery.

Autophagy, a fundamental cellular process for the degradation and recycling of cytoplasmic components, is essential for maintaining cellular homeostasis and adaptive responses to stress (Ajoolabady et al., 2021). Emerging evidence highlights a complex and often paradoxical role for autophagy in stroke, where its influence on neuronal survival is highly context-dependent (Ajoolabady et al., 2021; Wang X. et al., 2021). Although controlled autophagic flux can exert neuroprotective effects, excessive or dysregulated activation may exacerbate brain injury (Ajoolabady et al., 2021; Wang X. et al., 2021). This review synthesizes the evolving understanding of autophagy in stroke, moving from basic mechanisms to evaluating potential therapeutic targets and the challenges of clinical translation in the pursuit of precision stroke medicine.

## 2 Mechanisms of autophagy in stroke

Autophagy is a highly conserved cellular degradation process that maintains homeostasis by removing damaged organelles and misfolded proteins. This process is mediated by specific autophagy-related proteins and involves the formation of *de novo* bilayer membranes derived from the endoplasmic reticulum or Golgi complex (Lei et al., 2021). Three primary types of autophagy exist in mammals: microautophagy, macroautophagy, and chaperone-mediated autophagy (Mizushima, 2018). Macroautophagy is the most prevalent and is extensively studied in the context of cerebral ischemia.

**Figure 1:**
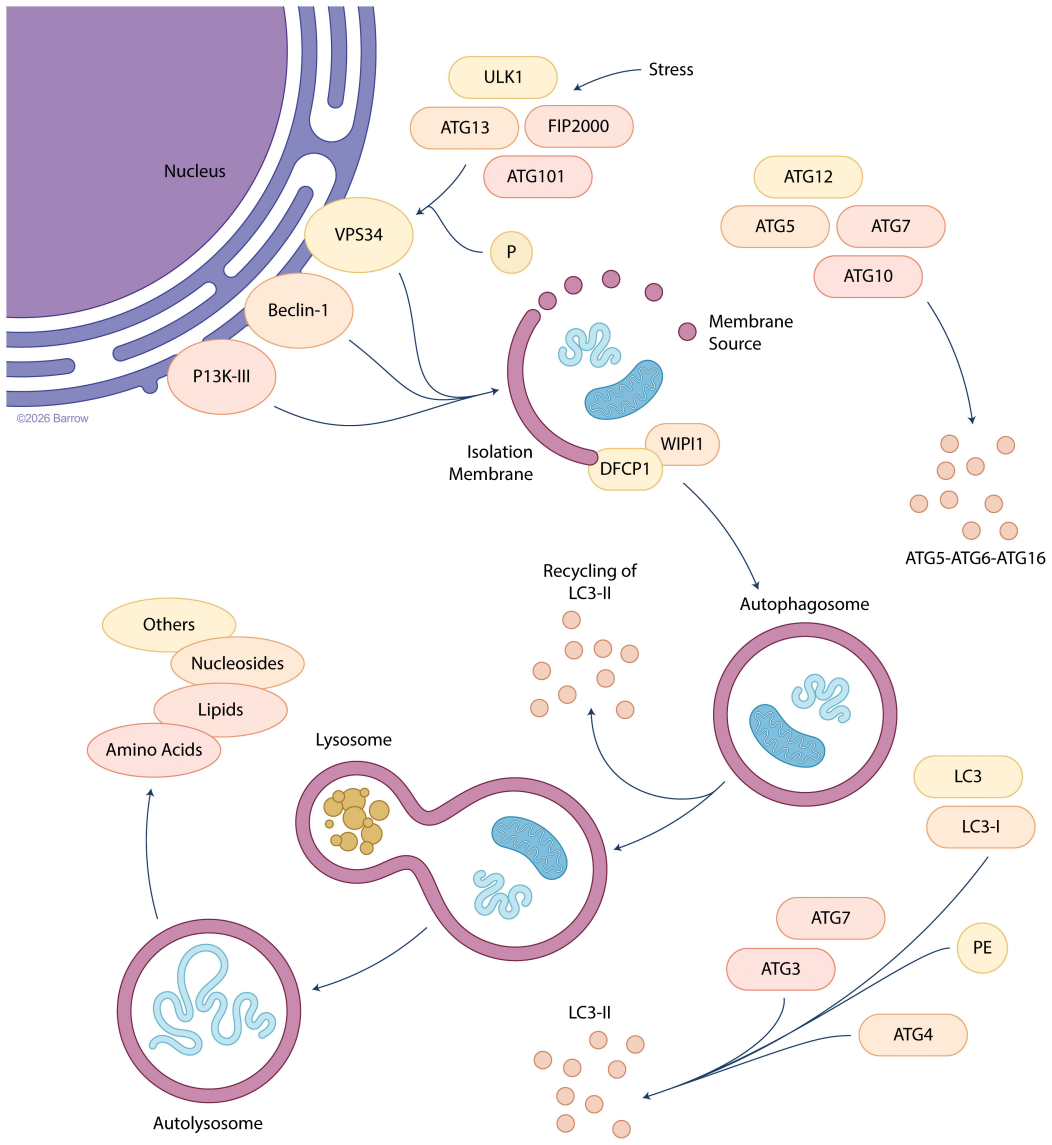
Molecular machinery of macroautophagy. Schematic illustration of the canonical pathway of macroautophagy, a cellular recycling process activated by stressors such as cerebral ischemia. Activation of the ULK1 complex (ULK1, ATG13, FIP200, and ATG101) in response to cellular stress phosphorylates and activates the VPS34 (phagophore). Isolation membrane elongation is mediated by ubiquitin-like conjugation pathways involving ATG12, ATG5, ATG7, and ATG10, which promote formation of the ATG5-ATG6-ATG16 complex, and the second conjugates LC3 to PE, yielding membrane-bound LC3-II. The incorporation of LC3-II facilitates the closure of the isolation membrane into a mature autophagosome, which subsequently fuses with a lysosome to form an autolysosome, where its cargo is degraded into reusable biomolecules. ATG, autophagy-related protein; DFCP1, double FYVE domain-containing protein 1; FIP200, FAK family kinase-interacting protein at 200 kDA; LC3, light chain 3; P, phosphorylation; p53, tumor protein 53; PE, phosphatidylethanolamine; PI3K, phosphatidylinositol 3-kinase; ULK1, Unc-51-like autophagy-activating kinase; VPS34, vacuolar protein sorting 34; WIPI1, WD repeat domain phosphoinositide-interacting protein 1. *Used with permission from Barrow Neurological Institute, Phoenix, Arizona*.

Macroautophagy follows a strictly regulated 4-stage progression: initiation, nucleation, maturation, and lysosomal fusion or degradation (Figure 1). Upon initiation, cytoplasmic cargo is encapsulated within a double-membrane vesicle known as an autophagosome. This vesicle then fuses with a lysosome to form an autolysosome, where acidic hydrolases degrade the sequestered components into reusable biomolecules (Su et al., 2022).

As postmitotic cells, neurons are particularly susceptible to the accumulation of protein aggregates and mitochondrial dysfunction, making this degradative pathway a critical determinant of neuronal fate (Gabryel et al., 2012). After an ischemic event, cellular stressors, including energy depletion, oxidative stress, and inflammation, trigger autophagic signaling (Lu et al., 2022). However, the functional outcome of this activation is not uniform; rather, it varies with the degree of regulation, intensity, and temporal stage of the injury (Tian et al., 2024). This duality, transitioning from a survival mechanism to a death-promoting pathway, is governed by a delicate homeostatic threshold that defines the Goldilocks zone of therapeutic intervention.

## 3 Molecular pathways regulating autophagy in stroke

**Figure 2:**
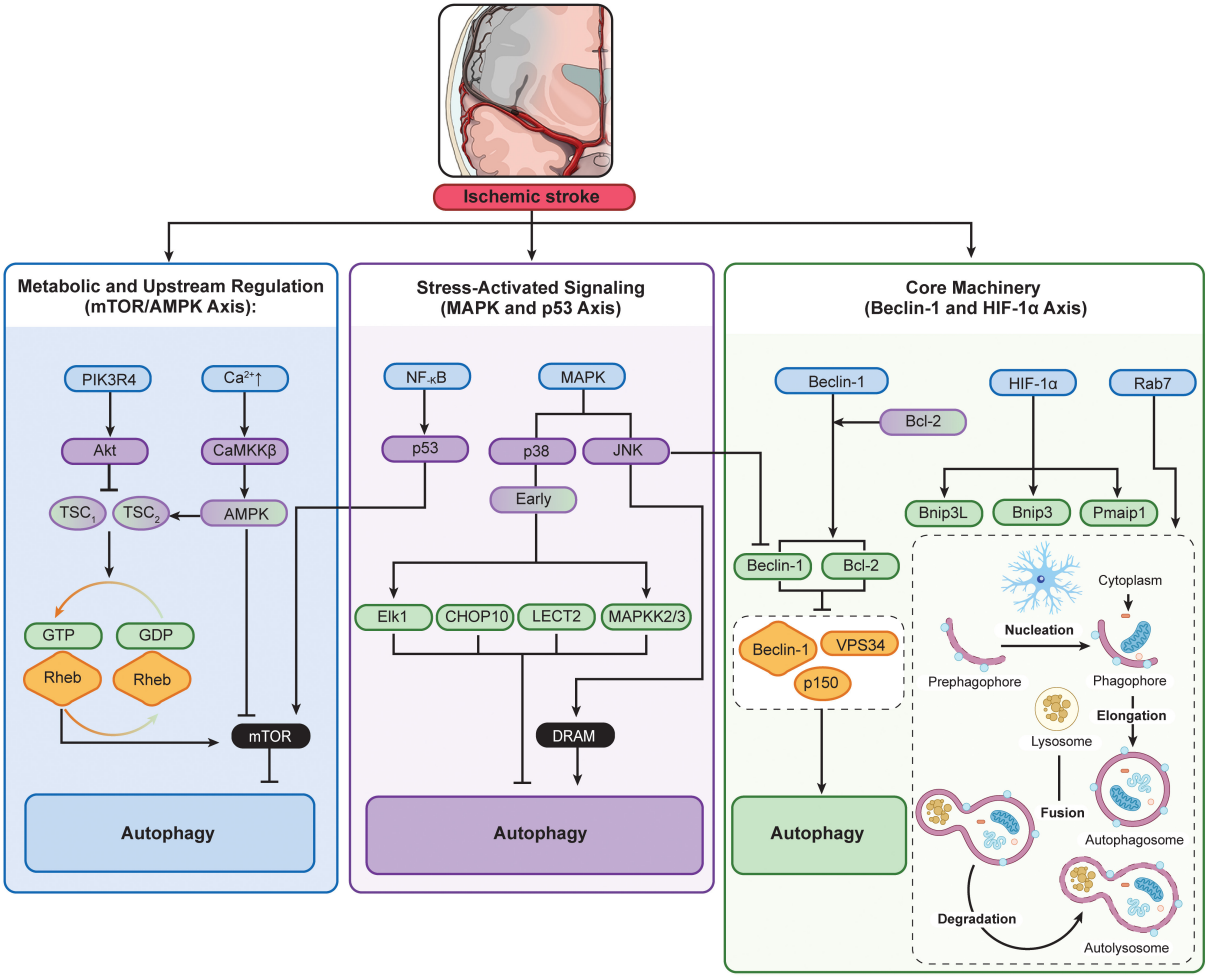
Key signaling pathways regulating autophagy in cerebral ischemia. Cerebral ischemia triggers a complex interplay of signaling pathways that regulate autophagy. The Akt and p53 pathways converge on mTOR, which acts as a primary inhibitor of autophagy. In contrast, AMPK, activated by cellular energy deficits, counteracts mTOR to induce autophagy. Concurrently, JNK activation promotes autophagy by disrupting the inhibitory Bcl-2–Beclin-1 complex, and hypoxia-driven hypoxia-inducible factor 1-alpha stimulates the process through effectors such as Bcl-2/adenovirus E1B 19-kDa interacting protein 3. Finally, Rab7 regulates the crucial late stage of autophagosome-lysosome fusion. These pathways collectively shape the dynamic autophagic response, determining whether the outcome is protective or detrimental. Akt, protein kinase B; AMPK, AMP-activated protein kinase; Bcl-2, B-cell lymphoma 2; Bnip3, BCL2 interacting protein 3; CaMKKα, calmodulin-dependent protein kinase kinase α; CHOP10, C/EBP homologous protein; DRAM, damage-regulated autophagy modulator; Elk1, ETS Like-1 protein; GDP, guanosine diphosphate; GTP, guanosine-5'-triphosphate; HIF-1α, hypoxia-inducible factor 1-alpha; JNK, c-Jun N-terminal kinase; LECT2, leukocyte cell-derived chemotaxin-2; MAPK, mitogen-activated protein kinase; MAPKK, mitogen-activated protein kinase kinase; mTOR, mammalian target of rapamycin; NF-κB, nuclear factor kappa-light-chain-enhancer of activated B cells; p38, tumor protein 38; p53, tumor protein 53; PIK3R4, phosphoinositide 3-kinase regulatory subunit 4; Pmaip1, phorbol-12-myristate-13-acetate-induced protein 1; Rab7, Ras-related protein Rab-7A; Rheb, Ras homolog enriched in brain; TSC1, tuberous sclerosis protein 1; TSC2, tuberous sclerosis protein 2; VPS34, vacuolar protein sorting 34. *Used with permission from Barrow Neurological Institute, Phoenix, Arizona*.

The cellular response to ischemic injury involves complex modulation of autophagy through several core signaling pathways that dynamically determine whether the process promotes survival or death. Key regulators, including mammalian target of rapamycin (mTOR), AMP-activated protein kinase (AMPK), mitogen-activated protein kinase (MAPK), Beclin-1, p53, and Rab7, govern this process (Figure 2).

As the master regulator of cellular metabolism, mTOR acts as the primary off-switch (Levine and Kroemer, 2019; Trelford and Di Guglielmo, 2021; Villa-Gonzalez et al., 2022), blocking Unc-51-like autophagy-activating kinase 1 phosphorylation and autophagy initiation under normal, nutrient-rich conditions. However, ischemia downregulates mTOR activity *via* reduced phosphatidylinositol 3-kinase/Akt signaling, thereby activating autophagy (Eskandari et al., 2021; Wang Y. et al., 2021). Contemporary literature emphasizes that mTOR signaling intersects significantly with metabolic stress and the survival of the neurovascular unit; for instance, its inhibition is necessary to trigger the neuroprotective clearance of damaged organelles, yet sustained suppression may inadvertently interfere with the M2 microglial polarization required for subacute tissue repair (Melanis et al., 2023; Villa-Gonzalez et al., 2022). Understanding this hierarchy is essential for clinical translation, because mTOR-targeted therapies must be precisely timed to avoid interfering with essential protein synthesis during the recovery phase.

Conversely, AMPK is activated by cellular energy depletion, specifically a high AMP-to-ATP ratio, and promotes autophagy by both inhibiting mTOR and directly phosphorylating Unc-51-like autophagy-activating kinase 1, a process that contributes to preconditioning neuroprotection (Jiang et al., 2018; Tian et al., 2024). Integrating these metabolic signals with stress responses, the MAPK family, particularly p38 MAPK and JNK signaling, modulates the Beclin-1/Bcl-2 complex (Wang X. et al., 2021). Under stress, JNK-mediated phosphorylation of Bcl-2 causes it to dissociate from Beclin-1, freeing Beclin-1 to initiate autophagosome nucleation (Luo et al., 2024; Lv et al., 2025), although the subsequent cleavage of Beclin-1 by caspase-3 can irrevocably shift the balance from cytoprotective autophagy toward apoptosis (Li H. et al., 2021). This progression leads to the maturation phase, in which the conversion of light chain 3 (LC3)-I to LC3-II serves as a definitive hallmark of autophagosome formation. Although its presence often correlates with the clearance of damaged mitochondria (Lv et al., 2025), excessive LC3-II accumulation can lead to energy depletion and autophagic stress (Fu et al., 2022).

Finally, the GTPase Rab7 regulates the essential process of autophagosome-lysosome fusion; dysfunction in this final degradative step leads to the accumulation of stalled autophagic vesicles, resulting in significant neurotoxicity (Wang X. et al., 2021).

## 4 Autophagy in innate and adaptive immune cells and stroke immunopathology

Autophagy serves as a conserved homeostatic link between cellular stress responses and inflammatory signaling, functioning as a mechanistic bridge between immune activation and ischemic brain injury (Levine and Kroemer, 2019; Mo et al., 2020; Trelford and Di Guglielmo, 2021). Neuroinflammation is a major contributor to secondary injury after ischemic stroke; autophagy intersects with these inflammatory pathways by influencing cytokine-associated signaling and inflammasome-related injury cascades described in experimental stroke models (Hu et al., 2022; Luo et al., 2024; Mo et al., 2020; Yuan et al., 2024).

In innate immune cells relevant to stroke pathology, including microglia and infiltrating macrophages, autophagy regulates inflammatory output by coupling metabolic stress to organelle quality control. Specifically, mitochondrial homeostasis managed by autophagy influences reactive oxygen species (ROS)–mediated amplification of inflammatory injury (Cobley et al., 2018; Lei et al., 2021; Mo et al., 2020; Shao et al., 2020). Conversely, impaired autophagic flux results in the accumulation of damaged organelles and enhanced cellular stress, which exacerbates inflammatory injury patterns in ischemia-reperfusion models (Fu et al., 2022; Lei et al., 2021; Mo et al., 2020).

Autophagy also supports the viability and stress tolerance of adaptive immune cells, such as lymphocytes, thereby influencing the magnitude and persistence of poststroke immune responses (Levine and Kroemer, 2019; Trelford and Di Guglielmo, 2021). Shared upstream regulators, including AMPK and mTOR, integrate energy sensing with inflammatory signaling, reinforcing the concept that autophagy regulation is relevant to both parenchymal injury and immune-mediated components of ischemic brain damage (Jiang et al., 2018; Lu et al., 2022; Trelford and Di Guglielmo, 2021).

Overall, available evidence supports viewing autophagy as a context-dependent modulator: when appropriately regulated, it helps constrain inflammatory injury, whereas its dysregulation contributes to persistent neuroinflammation and secondary tissue damage (Fu et al., 2022; Mo et al., 2020; Stanzione et al., 2024; Yuan et al., 2024).

## 5 Dual role of autophagy: from neuroprotection to neurotoxicity

This immunological modulation, specifically the autophagy-dependent shift toward an anti-inflammatory M2 phenotype, serves as a primary determinant of the broader functional paradox observed in stroke (Ajoolabady et al., 2021; Fu et al., 2022). Ultimately, the impact of autophagy on ischemic outcomes is governed by a quantitative balance rather than a qualitative contradiction. Therapeutic efficacy is contingent upon maintaining autophagic flux within a precisely calibrated homeostatic threshold, ensuring sufficient organelle clearance without crossing into the tipping point of maladaptive over-activation (Levine and Kroemer, 2019; Wang X. et al., 2021).

### 5.1 Neuroprotective role: early stage, controlled autophagy

In the hyperacute and early stages of stroke, moderate and well-regulated autophagy plays a neuroprotective role by mitigating apoptosis and secondary tissue damage (Mo et al., 2020). During this phase, autophagy facilitates the clearance of protein aggregates generated by ischemia-induced endoplasmic reticulum stress. Furthermore, it removes proapoptotic factors, such as Bax, Bim, and p53, through damage-regulated autophagy modulator 1–mediated mitochondrial regulation (Mao et al., 2022). Mitophagy, particularly through the PTEN-induced kinase 1–Parkin pathway, is essential for the selective removal of dysfunctional mitochondria before they can release cytochrome c and trigger apoptotic cascades (Shao et al., 2020; Zheng et al., 2023; Zille et al., 2019). The neuroprotective response also involves the active modulation of oxidative stress and neuroinflammation. The conversion of LC3-I to LC3-II and the activity of Beclin-1 promote a robust autophagic flux critical for neuronal survival (Cong et al., 2022; He et al., 2022). This flux further degrades oxidized lipids and promotes a phenotypic shift in microglia from proinflammatory M1 (secreting interleukin 6, interleukin 18, tumor necrosis factor-α) to anti-inflammatory M2 phenotypes that support tissue repair (Cobley et al., 2018; Lu et al., 2022).

**Figure 3:**
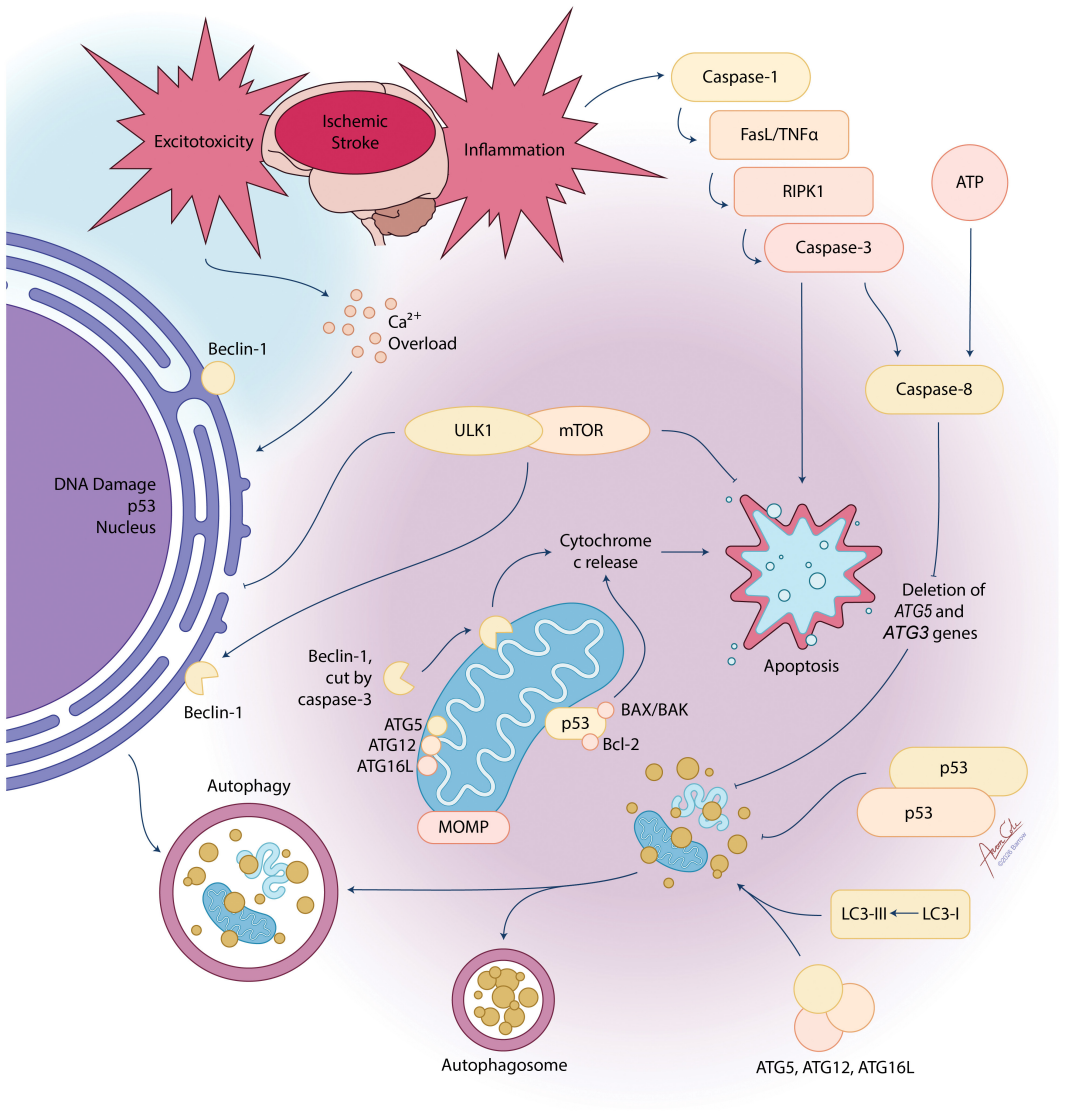
Molecular crosstalk between autophagy and apoptosis in the ischemic neuron. Schematic illustration of the intricate relationship between prosurvival autophagy and prodeath apoptosis after a cerebral ischemia. Neuronal fate is determined by a complex interplay of signaling cascades. Ischemic triggers such as excitotoxicity and neuroinflammation lead to severe intracellular stress, including Ca^2+^ overload and DNA damage, which activates the p53 pathway. Beclin-1 acts as a critical node; in its intact form, it promotes cytoprotective autophagy. However, inflammatory signaling and mitochondrial dysfunction can activate caspases, which cleave Beclin-1, abrogating its proautophagic function and tipping the cellular balance toward apoptosis. The apoptotic cascade involves MOMP, leading to cytochrome c release and caspase-driven cell death. ATP, adenosine triphosphate; Bcl-2, B-cell lymphoma 2; FasL, Fas ligand; MOMP, mitochondrial outer membrane permeabilization; mTOR, mammalian target of rapamycin; p53, tumor protein 53; RIPK1, receptor-interacting serine/threonine-protein kinase 1; TNFα, tumor necrosis factor alpha; ULK1, Unc-51-like autophagy-activating kinase. *Used with permission from Barrow Neurological Institute, Phoenix, Arizona*.

Extracellular regulation also plays a pivotal role because astrocytes and mesenchymal stem cells (MSCs) regulate neuronal autophagic activity. Astrocyte-derived exosomes have been shown to activate neuronal autophagy to reduce injury, whereas MSCs secrete paracrine factors that induce protective autophagy and suppress systemic inflammation (Deng et al., 2022; Guo et al., 2021; Stanzione et al., 2024). The interplay between autophagy and apoptosis during this early window is governed by shared molecular nodes, including Beclin-1, p53, and LC3-II, which collectively dictate the transition from cellular stress to recovery (Figure 3; Tian et al., 2024).

### 5.2 Neurotoxic role: prolonged, excessive autophagy

In the later stages of stroke or under severe ischemic conditions, prolonged or excessive autophagy becomes detrimental, contributing to neuronal death. Pharmacological studies with 3-methyladenine (3-MA) or chloroquine (CQ) demonstrate that suppressing overactive autophagy reduces ischemic damage (Ginet et al., 2014; Hou et al., 2019). Poststroke overexpression of Beclin-1 and LC3-II has been linked to disrupted autophagic flux, resulting in autophagosome accumulation and neurotoxicity (Lauro et al., 2015; Li et al., 2024). This pathology is exacerbated by impaired lysosomal fusion, oxidative stress–induced membrane damage, and accumulation of cargo adaptors such as p62/SQSTM1, which further inhibit cargo degradation, exacerbating injury (Li et al., 2024). Additionally, autophagy may trigger ferroptosis through ferritinophagy-mediated iron release, promoting lipid peroxidation and ROS production (Dou et al., 2023). Experimental data also show that suppressing mitophagy or *ATG*7 with exosomal microRNAs can attenuate neuronal loss (Yin et al., 2025). In this way, modulating excessive autophagy could offer therapeutic promise in limiting ischemia-reperfusion injury.

The transition from protective to toxic autophagy does not have a sharply defined timeline and is highly dependent on the severity of the initial ischemic insult (Ajoolabady et al., 2021; Wang X. et al., 2021). Generally, this shift is thought to occur over hours to days after the stroke. Although this dual role is observed in both ischemic and hemorrhagic stroke, the specific triggers and outcomes can differ (Zheng et al., 2023). For instance, the profound inflammatory response and blood-brain barrier (BBB) disruption in hemorrhagic stroke may accelerate the transition to detrimental, excessive autophagy (Fu et al., 2022; Zheng et al., 2023).

**Figure 4:**
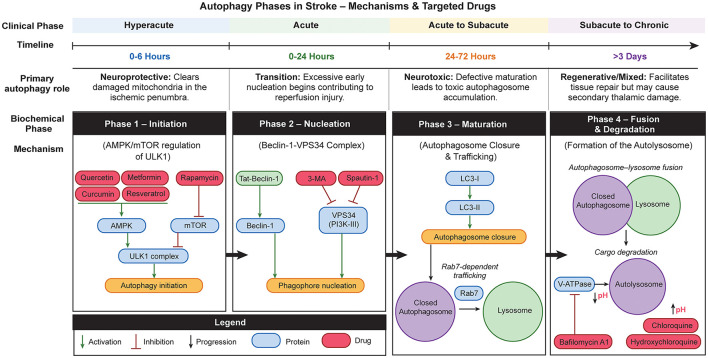
Autophagy phases in stroke-mechanisms and stage-specific therapeutic modulation. Schematic overview of the 4 phases of macroautophagy initiation (AMPK/mTOR–ULK1), nucleation (Beclin-1-VPS34), maturation (LC3 conversion and autophagosome trafficking), and fusion/degradation (lysosomal function). Representative drugs (*red*) are shown in red at their sites of action: quercetin, curcumin, metformin, and resveratrol targeting AMPK in the initiation phase; rapamycin targeting mTOR in the initiation phase; Tat-Beclin-1 promoting Beclin-1 activity and 3-MA and spautin-1 inhibiting VPS34 in the nucleation phase; and bafilomycin A1, chloroquine, and hydroxychloroquine targeting lysosomal acidification in the fusion/degradation phase. These phase-specific interventions highlight the therapeutic strategy of early autophagy induction for neuroprotection and later inhibition to limit excessive autophagy during reperfusion. 3-MA, 3-methyladenine; AMPK, AMP-activated protein kinase; LC3, light chain 3; mTOR, mammalian target of rapamycin; PI3K, phosphoinositide 3-kinase; Rab7, Ras-related protein Rab-7A; ULK1, Unc-51-like autophagy-activating kinase; V-ATPase, vacuolar-type ATPase; VPS34, vacuolar protein sorting 34. *Used with permission from Barrow Neurological Institute, Phoenix, Arizona*.

The outcome of autophagy activation in stroke is critically dependent on the timing, degree of activation, and molecular environment (Wang X. et al., 2021). In early ischemia, autophagy facilitates cellular adaptation to metabolic stress; however, in later stages, sustained activation can induce autophagic cell death. Oxidative stress, a central feature of stroke pathology, further modulates this duality by affecting mitochondrial function, lysosomal activity, and redox-sensitive signaling pathways (Trelford and Di Guglielmo, 2021). The dynamic interplay between autophagy and ROS, along with factors such as p53 and calcium overload, underscores the complexity of stroke pathogenesis and highlights the need for stage-specific therapeutic targeting (Tasdemir et al., 2008; Trelford and Di Guglielmo, 2021). As illustrated in Figure 4, the clinical success of targeting these pathways depends on a dynamic strategy: enhancing the homeostatic threshold in the hyperacute phase while suppressing maladaptive flux during the acute and subacute windows.

## 6 Stage-specific autophagy and therapeutic windows

The clinical translation of the molecular and immunological mechanisms, as well as potential therapeutic targets, are inextricably linked to the temporal evolution of ischemic injury, categorized into hyperacute, acute, subacute, and chronic phases. In the hyperacute phase (0–6 h), the brain harbors the ischemic penumbra, where moderate autophagy functions as a critical survival mechanism (Hong et al., 2023). During this window, ischemic preconditioning activates the AMPK-dependent pathway to enhance cellular resilience, while the ATM/CHK2/Beclin-1 axis helps neutralize oxidative stress to prevent penumbral tissue from transitioning into the necrotic core (Guo et al., 2020; Nishino et al., 2004). Consequently, the therapeutic goal in this phase is the induction of autophagy (phase 1: initiation, and phase 2: nucleation) using agents such as metformin, rapamycin, resveratrol, quercetin, curcumin, and trehalose, as well as the Tat-Beclin-1 peptide to bolster these endogenous neuroprotective responses (Carloni et al., 2010; Grewal et al., 2021; Khan et al., 2020; Tian et al., 2024).

As the injury progresses into the acute phase (6–24 h), the role of autophagy often becomes maladaptive, particularly during reperfusion injury. The sudden influx of oxygen and resulting oxidative stress can trigger excessive autophagic flux, which exacerbates BBB disruption and promotes autophagic cell death. In this stage, signaling through Nogo-A and its receptors can exacerbate neuronal loss, making targeted inhibition (phase 2: nucleation, and phase 4: fusion and degradation) with early-stage inhibitors, like 3-MA or spautin-1, and late-stage inhibitors, such as CQ, hydroxychloroquine (HCQ), or bafilomycin A1, a more effective strategy to mitigate secondary tissue damage (Gunn et al., 2018; Hong et al., 2023; Levine and Kroemer, 2019; Zhao et al., 2022).

In the subacute phase (24 h to several days) and extending into the chronic stage, autophagy transitions toward a regenerative role, intersecting with neuroinflammation and tissue repair (Guo et al., 2021; Stanzione et al., 2024). Although regulated autophagy is essential for the anti-inflammatory M2 microglial shift and supporting neurogenesis (Mo et al., 2020; Stanzione et al., 2024), chronic overactivation may contribute to secondary neurodegeneration and impaired functional recovery (Stanzione et al., 2024; Wang X. et al., 2021). Advanced strategies in these phases use dynamic modulators to fine-tune autophagic pathways, ensuring the process supports tissue repair rather than persistent neurodegeneration.

## 7 Therapeutic modulation of autophagy in stroke

The dual role of autophagy in ischemic injury has shifted the therapeutic focus toward stage-specific pharmacological strategies. Preclinical research has established that, although baseline autophagic flux is neuroprotective, excessive or prolonged activation during late-stage reperfusion contributes to programmed cell death (Menzies et al., 2017). This foundational understanding has prompted numerous studies evaluating the benefits of both pharmacological induction and inhibition. To date, these interventions have shown promising results across various preclinical stroke models, although translation to human clinical trials remains a significant hurdle (Liu S. et al., 2022; Parrella et al., 2020). A summary of representative pharmacologic and biologic strategies, including their molecular targets, mechanisms of action, and stage-specific relevance derived almost exclusively from experimental data, is presented in Table 1 (Chen et al., 2024; Gunn et al., 2018; Hadley et al., 2019; He et al., 2022; Hou et al., 2019; Jiang et al., 2018; Li Y. et al., 2021; Li H. et al., 2021; Liu S. et al., 2022; Lu et al., 2022; Menzies et al., 2017; Peng et al., 2024; Shen et al., 2023; Song et al., 2024; Wang et al., 2022; Wu et al., 2021; Xia et al., 2020; Yang et al., 2023; Yin et al., 2025; Zhang et al., 2020; Zhu et al., 2018).

**Table 1:** Pharmacologic and emerging therapies that target autophagy in ischemic stroke.

Category, therapy	Molecular target	Mechanism of action	Stroke stage and context
Inducers			
Rapamycin	mTOR	Suppresses mTORC1 to remove inhibition of ULK1 complex	Early ischemia: reduces infarct volume and BBB disruption (Li et al., 2014; Hadley et al., 2019)
Metformin	AMPK	Activates energy-sensing AMPK pathway	Early or metabolic stress: enhances clearance of damaged mitochondria (Lv et al., 2025; Zhu et al., 2018)
Resveratrol	SIRT1, AMPK	Activates SIRT1-dependent autophagy	Early ischemia: provides neuroprotection against oxidative stress (Liu S. et al., 2022; Wang et al., 2022)
Tat-BECN1	Beclin-1	Enhances autophagosome formation	Experimental models: directly promotes autophagic flux (He et al., 2022)
15.6-7.2,-26498pt Trehalose	AMPK, TFEB-associated lysosomal biogenesis	Activates master regulator of lysosomal biogenesis	Preclinical: promotes lysosomal biogenesis and enhances autophagic flux (Menzies et al., 2017; Wu et al., 2021)
Inhibitors			
3-Methyladenine (3-MA)	PI3K-III (VPS34)	Blocks early-stage autophagosome formation	Late reperfusion: prevents excessive autophagic cell death (Lu et al., 2022; Gunn et al., 2018)
Chloroquine, HCQ	Lysosome	Impairs autophagosome-lysosome fusion by raising pH	Late reperfusion: suppresses detrimental overactive autophagy (Zhang et al., 2020; Peng et al., 2024)
Bafilomycin A1	V-ATPase	Prevents fusion and degradation	Late stage: inhibits the final degradative step (Hou et al., 2019; Gunn et al., 2018)
15.6-7.2,-26498ptSpautin-1	Beclin-1	Promotes Beclin-1 degradation through inhibition of the deubiquitinases USP10 and USP13, thereby suppressing autophagy	Experimental ischemia models: autophagy inhibition associated with reduced neuronal injury (Lu et al., 2022; Gunn et al., 2018)
Advanced			
MSCs, exosomes	Multiple	Modulates autophagy and inflammatory signaling through paracrine and extracellular vesicle–mediated mechanisms	Preclinical models: enhances tissue repair, reduces neuroinflammation, and promotes functional recovery (Yin et al., 2025; Song et al., 2024; Shen et al., 2023; Chen et al., 2024; Li Y. et al., 2021; Xia et al., 2020; Yang et al., 2023)

Evidence is derived primarily from preclinical and experimental studies unless otherwise specified; clinical translation remains limited.

3-MA, 3-methyladenine; AMPK, AMP-activated protein kinase; BBB, blood-brain barrier; HCQ, hydroxychloroquine; MSC, mesenchymal stem cell; mTOR, mammalian target of rapamycin; mTORC1, mammalian target of rapamycin complex 1; pH, potential of hydrogen (measure of hydrogen ion concentration); PI3K, phosphatidylinositol 3-kinase; SIRT1, sirtuin 1; Tat-BECN1, transactivator of transcription human autophagy protein Beclin-1; TFEB, transcription factor E-binding; ULK1, unc-51-like autophagy activating kinase 1; USP10, ubiquitin-specific peptidase 10; USP13, ubiquitin specific protease 13; V-ATPase, vacuolar-type adenosine triphosphatase; VPS34, vacuolar protein sorting 34 (class III phosphatidylinositol 3-kinase).

### 7.1 Autophagy induction in stroke

#### 7.1.1 Rapamycin and mTOR Inhibitors

Rapamycin is one of the most well-studied autophagy inducers in the laboratory. The drug acts as a suppressor of mTORC1 signaling, thereby removing its inhibitory effect on autophagy, which helps reduce neuronal loss, BBB disruption, and inflammatory responses in experimental models (Lu et al., 2022; Zhang et al., 2024). Studies using a middle cerebral artery occlusion (MCAO) rat model revealed rapamycin's role in enhancing autophagic flux (Li et al., 2014). Other studies demonstrate that rapamycin enhances LC3-II expression, reduces infarct volume, and improves motor function in rodent models of ischemic stroke (Shi et al., 2021). However, some authors suggest that rapamycin's potent immunosuppressive effect could increase the risk of infections in stroke patients, preventing its use in clinical settings (Villa-Gonzalez et al., 2022).

#### 7.1.2 Natural compounds

Various studies have used natural compounds, such as resveratrol, curcumin, and quercetin, to explore their autophagy-inducing properties *in vitro* and *in vivo* (Grewal et al., 2021; Khan et al., 2020; Zhu et al., 2022). In rodent stroke models, resveratrol, a polyphenol found in red wine, showed potential neuroprotective effects, because it activates sirtuin 1–dependent autophagy and reduces ischemic injury. Curcumin is thought to enhance autophagy *via* the activation of the AMPK/mTOR pathway, thus reducing cerebral edema and inflammation in experimental stroke (Khan et al., 2020; Zhu et al., 2022). Similarly, autophagy is activated by quercetin, a flavonoid found in some fruits and vegetables that protects cultured neurons against oxidative stress (Grewal et al., 2021).

#### 7.1.3 Other small molecules and peptides

In addition to natural compounds and mTOR inhibitors, several small molecules and peptides have been identified as autophagy inducers with neuroprotective potential in preclinical stroke research. For example, trehalose activates transcription factor EB, a master regulator of autophagy and lysosomal biogenesis (Wu et al., 2021). In experimental stroke models, the Beclin-1–derived peptide Tat-Beclin-1 enhances autophagosome formation and reduces infarct volumes (He et al., 2022), whereas metformin activates AMPK in diabetic animal models, conferring neuroprotection (Jiang and Liu, 2020).

#### 7.1.4 Other autophagy inducers

Other autophagy inducers have gained attention as potential therapeutic agents for stroke, given their ability to promote cellular repair, reduce oxidative stress, and enhance neuroprotection (Ajoolabady et al., 2021). The synergistic potential of normobaric oxygen (NBO) therapy combined with autophagy modulation has been explored, with a suggestion that oxygenation therapy may help optimize the beneficial effects of autophagy (Wang M. et al., 2021). However, some have reported that hypoxia-induced autophagy contributed to neuronal cell death rather than survival, indicating that excessive or dysregulated autophagy can accelerate ischemic injury (Yang et al., 2014). These conflicting findings underscore that therapeutic autophagy induction requires precise modulation, because it may be beneficial at certain stages of stroke but harmful at others. More studies are needed to define the precise therapeutic windows in which autophagy activation can enhance stroke recovery and not exacerbate cell death.

### 7.2 Autophagy inhibition in stroke

Research has suggested autophagy inhibition as an alternative to prevent neuronal apoptosis and secondary brain damage, which has been widely linked to excessive activation of autophagy in laboratory settings (Carloni and Balduini, 2020; Menzies et al., 2015). In experimental studies, prolonged autophagy activation can lead to excessive degradation of functional organelles, promoting neuronal death, which is distinct from apoptosis and necrosis (Denton and Kumar, 2019; Luo et al., 2020; Wang and Xu, 2020; Wang X. et al., 2021). Furthermore, overactive autophagy associated with prolonged ischemia-reperfusion injury models results in neuroinflammation exacerbation and BBB breakdown (Gao et al., 2021). Of note, autophagy inhibition was particularly effective in female stroke models, highlighting the need for personalized treatment strategies that consider sex-based variations in therapeutic efficacy (Patrizz et al., 2021). Thus, modulating autophagy could be essential in later stages of stroke, when excessive autophagy activity contributes to neurodegeneration (Li X. et al., 2022).

#### 7.2.1 Chloroquine and hydroxychloroquine: late-stage autophagy inhibitors

CQ and HCQ inhibit autophagy at the lysosomal degradation stage by impairing autophagosome-lysosome fusion (Levine and Kroemer, 2019). In an experimental ischemia-reperfusion injury model, CQ administration reduced infarct volume and improved motor recovery (Zhang et al., 2020). Autophagy inhibition properties of HCQ have been tested in neurodegenerative diseases; however, there are currently no clinical trials for stroke (Peng et al., 2024).

#### 7.2.2 3-methyladenine: early-stage autophagy inhibitor

3-MA is a phosphatidylinositol 3-kinase inhibitor that blocks autophagy at an early stage, preventing the formation of autophagosomes (Hou et al., 2019). 3-MA in MCAO models helps reduce infarct volume and decrease autophagy-related neuronal loss (Su et al., 2014), and the combination of this molecule with other neuroprotective agents such as antioxidants demonstrates synergistic benefits (Ahmadzadeh et al., 2025).

#### 7.2.3 Other autophagy inhibitors

Several molecules have shown potential autophagy-inhibitory effects in stroke models. For example, bafilomycin A1 prevents autophagosome-lysosome fusion, and spautin-1, a selective Beclin-1–associated autophagy inhibitor, reduces neuronal apoptosis in ischemic models (Gunn et al., 2018; Zhao et al., 2022). In MCAO models, VX-765, a caspase-1 inhibitor, indirectly modulated autophagy *via* inflammasome suppression (Yuan et al., 2024). In addition, acupuncture has been explored as an alternative method to modulate autophagy, because it might inhibit mTOR-mediated excessive autophagy, thereby improving functional recovery (Liu H. et al., 2022).

### 7.3 Combined strategies and synergistic modulation

The therapeutic potential of autophagy modulation is best maximized when integrated into a multitargeted strategy rather than used as monotherapy. One area of growing interest is the combination of autophagy-targeting approaches with adjunctive therapies that influence ischemia–reperfusion biology. NBO therapy in experimental models reduces ischemia–reperfusion injury by suppressing early excessive autophagy and preserving BBB integrity; these mechanisms suggest that adjunctive modulation of autophagy may enhance tissue tolerance to ischemia-reperfusion injury (Wang M. et al., 2021).

Combination approaches that simultaneously target oxidative stress represent another rational strategy, because reactive oxygen species are major upstream triggers of autophagic dysregulation after ischemia. Preclinical studies indicate that pharmacologic autophagy inhibitors such as 3-MA, when used alongside antioxidant or multitarget neuroprotective interventions, enhance neuroprotective efficacy compared with single-pathway approaches (Ahmadzadeh et al., 2025; Liu S. et al., 2022; Lu et al., 2022). Such dual-pathway targeting may stabilize the balance between adaptive autophagy and the transition to apoptosis or autophagic cell death.

Novel regenerative strategies further illustrate the integration of pharmacologic and biologic approaches. MSC-based therapies dynamically modulate autophagy, promote neuronal survival, reduce neuroinflammation, and improve functional recovery (Chen et al., 2024; Shen et al., 2023; Song et al., 2024). Genetic or molecular priming of MSCs and the use of stem cell–derived extracellular vesicles or exosomal microRNAs may enhance therapeutic efficacy by enabling targeted regulation of autophagic pathways within the neurovascular unit (Li Y. et al., 2021; Xia et al., 2020; Yang et al., 2023). These multimodal strategies represent a promising translational direction aimed at achieving spatiotemporally precise modulation of autophagy in ischemic stroke. However, most combination autophagy-targeting strategies remain at the preclinical stage, and clinical validation is limited.

### 7.4 Stem cell therapy and autophagy modulation in stroke

Stem cell therapy is an emerging strategy for stroke recovery given its potential to promote autophagy modulation and influence cell survival, inflammation, and neuronal repair (Song et al., 2024). One recent study suggests that some stem cells promote autophagy to remove damaged mitochondria and support neuronal survival (Shen et al., 2023). On the other hand, others demonstrated that MSCs downregulate overactive autophagy and reduce neuronal apoptosis (Chen et al., 2024). Moreover, stem cell therapy acts by influencing inflammation and immune response, which ultimately helps regulate microglial activation, reducing neuroinflammation and secondary brain injury (Liu W. et al., 2020).

Strategies to improve effectiveness of stem cell therapy have been explored. For example, GATA3 was shown to enhance bone marrow–derived MSC-mediated neuroprotection through autophagy modulation, indicating that genetic or pharmacological manipulation of autophagy could improve stem cell therapy efficacy (Li Y. et al., 2021). Extracellular vesicles secreted by MSCs derived from human induced pluripotent stem cells were found to reduce infarct size and prevent excessive autophagy, illustrating a potential noncellular approach to stem cell–based autophagy modulation (Xia et al., 2020). Exosomal microRNA, particularly miR-133a-3p, can provide neuroprotection by suppressing autophagy overactivation, presenting an alternative to direct stem cell transplantation (Yang et al., 2023). By optimizing autophagy regulation in stem cell therapy, researchers may develop more effective regenerative treatments for stroke recovery.

## 8 Integration with acute reperfusion therapies

A critical frontier in stroke research is the synergistic integration of autophagy modulation with established acute therapies, namely intravenous thrombolysis and mechanical thrombectomy. Although intravenous thrombolysis and mechanical thrombectomy are highly effective at restoring macrovascular patency, the sudden return of blood flow to ischemic tissue often triggers ischemia-reperfusion injury, a process driven by oxidative stress and mitochondrial dysfunction (Ayomide et al., 2025). Preclinical evidence suggests that the neuroprotective efficacy of reperfusion could be significantly enhanced by the co-administration of autophagy modulators (Tang et al., 2025). For example, suppressing the early, excessive autophagic flux that occurs immediately upon recanalization, using agents like 3-MA (Zhang et al., 2021), has been shown to preserve BBB integrity and reduce the risk of hemorrhagic transformation, a common complication of intravenous thrombolysis.

Furthermore, the timing of these adjuncts could be tailored to the specific procedural stage of the intervention. Hyperacute induction of autophagy during the prehospital or drip phase of thrombolysis could prime neurons to survive bioenergetic failure, and acute inhibition during or immediately after mechanical clot retrieval could mitigate the metabolic surge of reperfusion (Tang et al., 2025). Emerging data also suggest that combining NBO therapy with autophagy inhibitors during the periprocedural window of mechanical thrombectomy may optimize tissue salvage in the penumbra by stabilizing mitochondrial membranes (Wang M. et al., 2021). As mechanical thrombectomy continues to expand its therapeutic window, the integration of these biologic adjuncts offers a promising strategy to bridge the current gap between successful vessel recanalization and meaningful functional recovery.

## 9 Clinical evidence and translational challenges

Despite a robust body of preclinical evidence, the transition of autophagy-modulating therapies from the laboratory to the bedside remains in its infancy (Ajoolabady et al., 2021; Stanzione et al., 2024; Wang X. et al., 2021). This gap can be understood through the lens of prior neuroprotection trials, which have consistently faced failures in clinical translation (Gunn et al., 2018; Menzies et al., 2017). Over the past 3 decades, more than 1,000 experimental agents showed preclinical promise, yet nearly all failed in phase II and III trials (Menzies et al., 2017). These failures have been largely attributed to one-size-fits-all approaches that failed to account for patient heterogeneity and narrow therapeutic windows (Menzies et al., 2017; Parrella et al., 2020). This discrepancy is further driven by reliance on young, healthy MCAO rodent models that do not reflect the age-related comorbidities prevalent in human stroke populations (Parrella et al., 2020). Furthermore, biomarkers such as LC3-II and p62 have not translated into validated clinical tools for real-time decision-making, limiting the ability to define therapeutic windows in patients (Klionsky et al., 2021; Stanzione et al., 2024).

Direct evidence of autophagy in the human brain after stroke is limited but insightful (Stanzione et al., 2024). Postmortem analyses have identified significant increases in p62 and LC3 accumulation in ischemic regions, suggesting that impaired autophagic flux is a genuine feature of human pathology (Stanzione et al., 2024). In living patients, cerebrospinal fluid and serum levels of Beclin-1 and LC3B are elevated following acute ischemic stroke and correlate with larger infarct volumes and higher National Institutes of Health Stroke Scale scores (Stanzione et al., 2024). Emerging research into biofluid markers and molecular imaging, such as positron emission tomography or magnetic resonance imaging with nanoparticle-based probes, represents a promising frontier for visualizing autophagic vesicles *in vivo* to guide patient selection (Klionsky et al., 2021).

Although no drug is currently approved specifically for autophagy modulation in stroke, repurposed agents are under investigation (Ajoolabady et al., 2021; Wang X. et al., 2021). The most concrete evidence involves metformin, an AMPK activator; a specific trial found that metformin reduced poststroke cognitive impairment, potentially by enhancing the clearance of damaged mitochondria (Zhu et al., 2018). However, systemic delivery poses safety concerns; potent inducers like rapamycin are heavily immunosuppressive, heightening the risk of poststroke infections, and broad inhibitors could interfere with homeostatic functions in nonischemic tissues (Ajoolabady et al., 2021; Hadley et al., 2019). Additionally, many promising molecules struggle with effective BBB penetration at therapeutic concentrations (Ajoolabady et al., 2021; Stanzione et al., 2024). Moving forward, clinical success will likely require spatiotemporally precise interventions, such as exosomal delivery or stem cell therapies, tailored to the individual's genetic profile and ischemic stage (Shen et al., 2023; Song et al., 2024).

## 10 Investigative trends in autophagy and stroke

**Figure 5:**
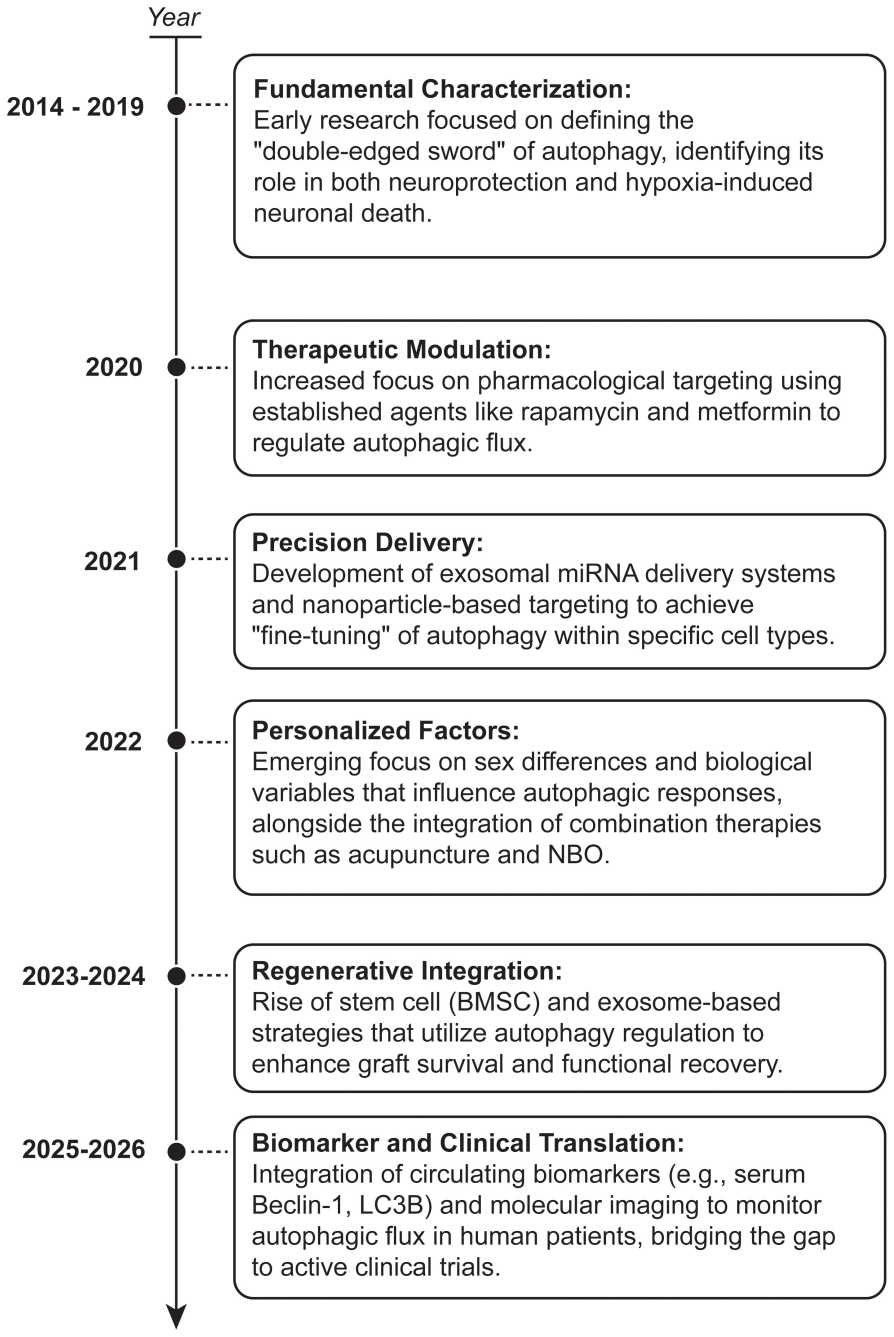
Timeline of research trends in autophagy and stroke. Progression of autophagy-based stroke therapy research over the past decade showing evolution from early pre-2020 studies focused on basic mechanisms and neurotoxicity, to a more recent focus on therapeutic modulation with compounds such as rapamycin (2020), and finally to advanced regenerative strategies using stem cells, exosomes, and microRNA (miRNA)–based therapies to achieve precision modulation (2021-present). BMSC, bone marrow mesenchymal stem cell; NBO, normobaric oxygen. *Used with permission from Barrow Neurological Institute, Phoenix, Arizona*.

The evolution of stroke-related autophagy research has progressed through three key phases, reflecting a significant shift from basic mechanistic discovery toward a nuanced, spatiotemporal understanding of the Goldilocks zone (Figure 5). Pre-2020 studies primarily investigated basic mechanisms, revealing autophagy's dual role in neuroprotection and hypoxia-induced neuronal death (Yang et al., 2014). These foundational years established the core molecular machinery of the autophagic lifecycle in ischemic models, utilizing broad pharmacological agents to demonstrate how autophagy could be either a lifeline for penumbral survival or a driver of autophagic cell death.

By 2020, the landscape transitioned into the targeted modulation and precision delivery phase. This period saw an increased focus on pharmacological targeting using established agents like rapamycin and metformin to regulate autophagic flux. This was followed in 2021 by the rise of precision delivery, characterized by the development of exosomal microRNA delivery systems and nanoparticle-based targeting to achieve “fine-tuning” of autophagy within specific cell types. In 2022, researchers began prioritizing personalized biological variables, such as sex differences that influence autophagic responses, alongside the integration of combination therapies, including acupuncture and NBO.

From 2023 to 2024, the field entered the regenerative integration phase, marked by the rise of stem cell (BMSC) and exosome-based strategies that utilize autophagy regulation to enhance graft survival and functional recovery. Currently, in 2025 and 2026, the focus has shifted toward biomarker and clinical translation. The present era emphasizes the integration of circulating biomarkers, such as serum Beclin-1 and LC3B, and molecular imaging to monitor autophagic flux in human patients. By bridging the gap to active clinical trials, this current frontier aims to identify the exact molecular switches that can be manipulated to extend the therapeutic window and ensure that successful vessel recanalization translates into meaningful functional neurological recovery.

## 11 Discussion and future directions

Collectively, evidence suggests that the effects of autophagy in ischemic stroke are time- and severity-dependent, with protective and harmful outcomes reported across experimental models. This dual, stage-dependent behavior represents a major challenge for the development of autophagy-targeted therapies. A critical analysis of the literature reveals that the functional outcome of autophagy is not intrinsic to the process itself but is instead dictated by the precise spatiotemporal context of the ischemic injury, the intensity of its induction, and the integrity of downstream molecular machinery. This variability suggests that therapeutic strategies should prioritize dynamic, stage-specific modulation rather than uniform induction or inhibition of autophagy.

The switch from neuroprotection to neurotoxicity appears to be governed by a delicate homeostatic threshold within the salvageable ischemic penumbra. In the initial hours after occlusion, a regulated increase in autophagy is a critical adaptive response to bioenergetic failure and proteotoxic stress. By facilitating the selective clearance of damaged mitochondria and misfolded protein aggregates, this early autophagic flux mitigates the activation of intrinsic apoptotic pathways and preserves neuronal viability. Key molecular events, including the balanced regulation of Beclin-1 and the lipidation of LC3, are indispensable for this prosurvival function. Conversely, under conditions of severe or prolonged ischemia and subsequent reperfusion, this adaptive process becomes dysregulated. The resulting maladaptive autophagy contributes directly to secondary neuronal demise through autophagic cell death, the toxic accumulation of stalled autophagosomes due to impaired lysosomal fusion, and the exacerbation of neuroinflammation.

This mechanistic duality creates a significant therapeutic conundrum. Preclinical studies have convincingly demonstrated the neuroprotective efficacy of both pharmacological autophagy inducers and inhibitors. Molecules such as rapamycin and resveratrol can salvage neurons by augmenting the beneficial, homeostatic aspects of autophagy, whereas 3-MA and CQ can prevent neuronal loss by blocking the deleterious effects of excessive, late-stage autophagy. These results underscore the most formidable barrier to clinical translation: defining the therapeutic window for targeting autophagy. The optimal intervention likely requires dynamic modulation, potentially enhancement in the hyperacute phase followed by suppression during reperfusion. Such a strategy remains clinically challenging, magnified by patient-specific variables, such as the sex-specific differences in autophagic responses observed in experimental models, which highlights the need for personalized therapeutic approaches.

Furthermore, a substantial gap remains between promising preclinical findings and clinical translation. To understand the translational potential of autophagy modulation, it must be viewed through the lens of prior neuroprotection trials. For 3 decades, over 1,000 stroke therapies failed to transition from laboratory models to human clinical success (Menzies et al., 2017). Standardized treatment models ultimately doomed these efforts by failing to account for stroke diversity and strict intervention timelines (Menzies et al., 2017; Parrella et al., 2020). Autophagy-targeted strategies face these same historical risks, particularly the model-patient discrepancy where young, healthy animals do not reflect the aged, comorbidity-burdened human population. Consequently, biomarkers such as LC3-II and p62 have not been validated as dynamic clinical tools to guide therapeutic decision-making in real-time.

However, autophagy modulation offers a distinct advantage over previous neuroprotective attempts by targeting a dynamic cellular process rather than a single static receptor. Earlier trials often focused on blocking a single pathway of the ischemic cascade, whereas autophagy modulation involves a broad homeostatic response that intersects with apoptosis, inflammation, and metabolic recovery. The future of this field may lie in moving beyond global induction or inhibition and toward next-generation neurotherapeutics capable of precision modulation.

The most promising strategies may involve targeting upstream master regulators of the autophagic network, such as the mTOR and AMPK signal transduction cascades, to achieve a more nuanced control over the process. Contemporary evidence underscores that, rather than merely acting as a binary switch for vesicle initiation, the poststroke modulation of the mTOR pathway serves as a master regulatory axis dictating the complex cross-talk between neuronal survival, glial scar formation, and downstream neuroinflammation (Melanis et al., 2023). Bi-directional manipulation of this cascade—repressing mTOR hyperacutely to clear cytotoxic aggregates, yet restoring its activity during the subacute phase to promote axonal sprouting and myelination—offers a sophisticated framework for precision neurotherapeutics.

Concurrently, regenerative medicine could offer a paradigm shift. Stem cell–based therapies, particularly BMSCs, appear to function as intelligent, endogenous modulators capable of dynamically adjusting the autophagic and neuroinflammatory milieu of the injured brain to promote repair. Engineered extracellular vesicles and microRNA-based approaches may enable more localized pathway modulation, bridging the gap to active clinical trials. By learning from the failures of the past, specifically by utilizing narrow, stage-specific windows and integrating these biologic adjuncts with mechanical thrombectomy, autophagy research has the potential to bypass the pitfalls that stalled earlier neuroprotective agents. The future of the field lies, not in a single-target pharmacologic strategy, but in the precision-timed regulation of autophagic flux alongside standard-of-care reperfusion.

## 12 Conclusion

The studies synthesized in this review confirm that the evolution of stroke research has reached a critical juncture where the focus must shift from simply restoring blood flow to preserving the delicate molecular architecture of the brain. Autophagy stands at the center of this transition, representing a fundamental homeostatic engine that can either drive recovery or accelerate destruction. This dual, stage-dependent behavior underscores that the therapeutic potential of autophagy is not a static property but a dynamic, spatiotemporal function of the ischemic process. Success in this field requires a shift toward a model of precision modulation.

By integrating established signaling switches, namely the mTOR and AMPK axes, with next-generation delivery systems like BMSC-derived exosomes, we can begin to fine-tune the autophagic lifecycle within specific stages of stroke evolution. This approach, paired with the development of circulating biomarkers like serum Beclin-1 and LC3B, offers a viable path to overcome the historical failures in clinical translation that have stalled prior neuroprotective efforts. As we align these biological strategies with current standards of care, such as mechanical thrombectomy, the goal is no longer just vessel recanalization, but true neurorestoration. Ultimately, mastering the Goldilocks zone of autophagy represents the next great frontier in stroke neurology, promising a future where molecular intervention and clinical precision unite to restore function and hope to patients worldwide.
